# The Mechanism of Layer Stacked Clamping (LSC) for Polishing Ultra-Thin Sapphire Wafer

**DOI:** 10.3390/mi11080759

**Published:** 2020-08-06

**Authors:** Zhixiang Chen, Linlin Cao, Julong Yuan, Binghai Lyu, Wei Hang, Jiahuan Wang

**Affiliations:** Ultra-Precision Machining Centre, College of Mechanical Engineering, Zhejiang University of Technology, Hangzhou 310014, China; czxiangcool@126.com (Z.C.); caolinlin0626@126.com (L.C.); icewater7812@126.com (B.L.); whang@zjut.edu.cn (W.H.); wangjiahuan1994@foxmail.com (J.W.)

**Keywords:** layer stacked clamping (LSC), ultra-thin sapphire, adhesion force, double-sides polishing

## Abstract

Double-sides polishing technology has the advantages of high flatness and parallelism, and high polishing efficiency. It is the preferred polishing method for the preparation of ultra-thin sapphire wafer. However, the clamping method is a fundamental problem which is currently difficult to solve. In this paper, a layer stacked clamping (LSC) method of ultra-thin sapphire wafer which was used on double-sides processing was proposed and the clamping mechanism of layer stacked clamping (LSC) was studied. Based on the rough surface contact model of fractal theory, combining the theory of van der Waals force and capillary force, the adhesion model of the rough surfaces was constructed, and the reliability of the model was verified through experiments. Research has found that after displacement between the two surfaces the main force of the adhesion force is capillary force. The capillary force decreases with the increasing of surface roughness, droplet volume, and contact angle. For an ultra-thin sapphire wafer with a diameter of 50.8 mm and a thickness of 0.17 mm, more than 1.4 N of normal adhesion force can be generated through the LSC method. Through the double-sides polishing experiment using the LSC method, an ultra-thin sapphire wafer with an average surface roughness (*R*_a_) of 1.52 nm and a flatness (PV) of 0.968 μm was obtained.

## 1. Introduction

Sapphire is one of the main materials of light emitting diode (LED) substrate due to its excellent material properties [1,2,3]. However, the sapphire material cannot have good thermal conductivity, and the temperature rise generated in the active area of the LED will cause the sapphire substrate to have a fatal effect on the light output characteristics and service life [4,5,6]. In order to improve the heat dissipation performance of the sapphire substrate, it is necessary to use a thinning process to thin the sapphire substrate [7] However, there is a surface damage layer on the thinned sapphire substrate [8,9], and the residual stress caused by the thinning process will cause the epitaxial wafer to bend and deform or even break [10], affecting the final yield. So, the ultra-precision processing of ultra-thin sapphire wafers is particularly important. Additionally, the capacity of sapphire wafers is an important factor restricting the expansion of the sapphire industry.

Planetary double-plane polishing is the preferred processing method for processing ultra-thin sapphire wafers. However, ultra-thin planetary wheels have problems of insufficient strength and rigidity, and paraffin bonding leads to high processing costs and low efficiency. So, the clamping method is a fundamental problem which is currently difficult to solve. Some scholars had conducted researches on using water as a medium to adsorb and clamp parts on two surfaces. However, the clamping mechanism was not deeply analyzed [11]. Since the surface of various materials is not absolutely smooth, roughness becomes important for the force between solid surfaces [12,13], and the expression model of rough surface morphology has been the basis for studying rough surface forces.

Fractal theory could properly characterize the surface roughness model, and it was widely used in the study of elastoplastic mechanical behavior of different material surfaces [14,15,16,17,18]. The rough surface profile is characterized by the G-W function which was established by Williamson and Greenwood [19]. Then, according to Hertz contact theory and elastic-plastic contact theory [20], the deformation states of different convex individuals on the rough surface were analyzed [16,21,22], moreover, the true contact area of the whole rough surface was constructed by using fractal theory [15,18,21].

For the solid-liquid-solid contact surface, the presence of the liquid medium changes the force between the two solids. Without considering the chemical action, the effect of the contact angle becomes particularly significant. An atomic force microscope (AFM) probe technique was widely used to study the relationship between contact angle and roughness [23,24]. Contact angle directly affects the height of capillary bridge between solid surfaces and the capillary force [25,26,27,28].

However, the above researches and analysis were mostly aimed at the results of the ideal rough plane. In this paper, an ultra-thin sapphire layer stacked clamping (LSC) method is proposed. The adhesion mechanism of the layered clamping is studied by constructing a rough surface contact model, and the effectiveness of the adhesion mechanism is verified through experiments. The double-plane polishing experiment shows that the LSC method can realize the double-sides processing of ultra-thin sapphire.

## 2. Principle of Layer Stacked Clamping (LSC)

**Figure 1:**
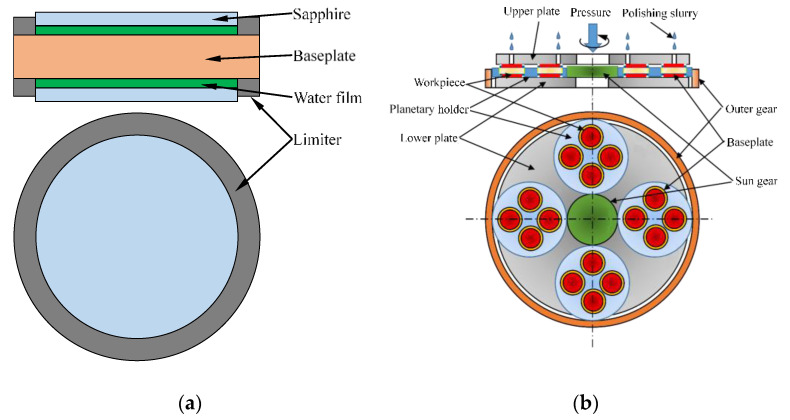
Schematic diagram of double-sides processing by layer stacked clamping (LSC) method: (**a**) principle of layer stacked clamping; (**b**) schematic diagram of layer stacked double-sides processing.

The principle of the layer stacked clamping (LSC) method is shown in Figure 1a. Two limiters are fixed on the upper and lower surfaces of the baseplate, and a hole is formed on the limiter that the workpiece can be placed in the limiter. The height difference between the surface of limiter and baseplate realizes the radial limit of the workpiece. When clamping the workpiece, water droplets are filled to form a water film between the two surfaces of baseplate and workpiece to realize the normal adhesion of the workpiece. So, the axial displacement of the workpiece is limited by the normal adhesion force. Since the two contacting surfaces cannot be completely smooth, there is a large amount of air between the two rough surfaces [11]. When two rough surfaces are in contact with each other without applying an external force, the adhesion force between the two surfaces is mainly based on van der Waals forces and capillary forces [29,30]. When the humidity is low, the force of the contact area is mainly van der Waals force which because the capillary force is greatly affected by the humidity of the air [31]. The real contact area is much smaller than the apparent contact area which is affected by the surface roughness [32,33,34]. Therefore, the force of van der Waals force on the workpiece is extremely small, the adhesion force between the surfaces is smaller than the weight of the workpiece, and the workpiece cannot be reliably adsorbed on the surface of baseplate. When the liquid is injected between the two surfaces to form a liquid film, the air between workpiece and baseplate is discharged by a liquid film. The adhesion force between two surfaces is greater than the weight of workpiece, and the workpiece can be adsorbed on the surface of baseplate in the normal direction. The limiter limits the radial movement of the workpiece so that the workpiece is firmly clamped on the fixture. At this time, a “workpiece–baseplate–workpiece” layered stacked clamping method is formed between the workpiece and the baseplate, the thickness of workpiece under the effect of the baseplate is equivalently thickened and can be clamped by ordinary planetary gears, as shows in Figure 1b. It is used for double-sides lapping and polishing of ultra-thin wafers.

## 3. Adhesion Mechanism of LSC

### 3.1. Fractal Theory of Rough Surface

According to the interface adhesion coefficient, there are normal adhesion force and friction force between them after two rough planes contact. When liquid is added between the two planes, the normal adhesion force increases. While the adhesion force is greater than the gravity of the plane part, the part can be firmly attached to another plane.

According to the G-W contact model established by Williamson and Greenwood, the curve profile of the rough surface can be obtained [19]:

(1) $$Z\left(x\right)=G^{D-1}\overset{\infty }{\underset{n=\mathrm{nl}}{\sum }}\frac{\cos\left(2\pi \gamma^{n}x\right)}{\gamma^{\left(2-D\right)n}},\;1<D<2,\gamma >1$$

where, *D* is fractal dimension of a surface profile, *G* is fractal roughness parameter, *γ* is scaling parameter for the Weierstrass–Mandelbrot function, *γ*^n^ determines the frequency spectrum of the surface roughness, the lowest frequency is related to the length *L* of the sample as *γ*^nl^ = 1/*L*.

In the actual contact of two micro-bulge, pressure change will cause the micro-bulge to change from point contact to surface contact. With the increase of contact area, the contact mode changes from plastic contact to elastic-plastic contact and then to elastic contact mode. The relationship between the deformation amount of the micro-bulge tip *δ* and the contact point area *a* as follows [21]:

(2) $$\delta =G^{D-1}a^{\frac{2-D}{2}}$$

The radius of curvature *R* of the micro-bulge is shown as the following formula [22]:

(3) $$R=\frac{a}{2\pi \delta }$$

According to Hertz contact theory [20], it can be obtained that the critical deformation amount *δ*_pe_ of the micro-bulge when it changes from elastic deformation to plastic deformation (Equation (4)):

(4) $$\delta_{\mathrm{pe}}={\left(\frac{\pi H_{b}}{2E^{*}}\right)}^{2}R$$

where, *H*_b_ is the hardness of material, E* is Elastic Modulus, $E^{*}=((1-v_{A}^{2})/E_{B}+(1-v_{B}^{2})/E_{B}{)}^{2}$, *v*_A_, *v*_B_ are the Poisson’s ratio of surface A and surface B, respectively, *E*_A_, E_B_ are the elastic modulus of surface A and surface B, respectively.

Through Equation (2) to Equation (4), the expression of critical contact area *a*_pe_ can be obtained as:

(5) $$a_{\mathrm{pe}}=G^{2}{\left(\frac{8E^{*2}}{\pi H_{b}{}^{2}}\right)}^{1/\left(D-1\right)}$$

### 3.2. Van der Waals Force Adhesion Model

The van der Waals attraction between atoms also exists on microscopic objects. It can be obtained by the sum of the forces between individual atoms or molecules of the object. The van der Waals force is sufficient to make the micro particles adhere to their matrix. The van der Waals force between the two surfaces varies with the distance between the two surfaces. The van der Waals force of the rough surface varies with the contour of the micro-bulge.

**Figure 2:**
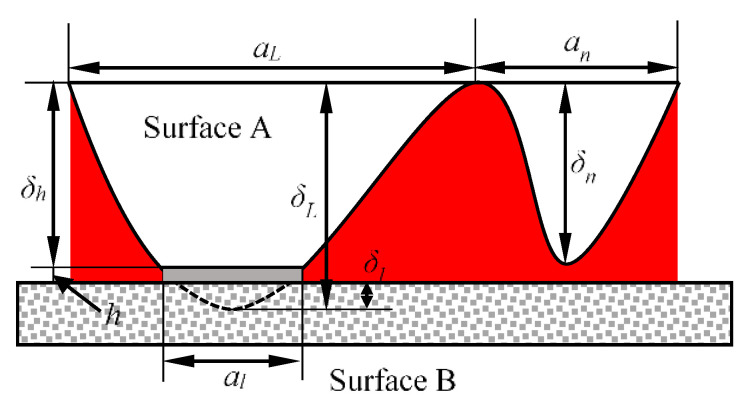
Rough surface contact.

As shown in the rough surface of Figure 2, a micro surface is formed when the micro-bulge is deformed by pressure. At this time, the micro-bulge is similar to a ball table, and the table height is:

(6) $$\delta_{h}=\delta_{L}-\delta_{l}$$

where *δ*_L_ is the height of the micro-bulge, and *δ*_l_ is the maximum deformation amount of the elastoplastic deformation of the micro-bulge.

Defining the height of a contact micro projection as *δ*_n_, its value is *δ*_n_ = *δ*_h_ + *δ*, and the area of the contact area is *a*, then the van der Waals work received by the micro projection which is based on the model of Israelachvili [35] is:

(7) $$W_{c}=\frac{2}{\left(n-2\right)\left(n-3\right)}\int_{0}^{\infty }\frac{J_{\mathrm{AWB}}\left(\left(2R-z-\delta \right)z-\frac{a}{\pi }\right)+\frac{J_{\mathrm{AB}}a}{\pi }}{{\left(h+z\right)}^{n-3}}dz$$

where *n* = 6, *δ* is the height of the deformation of the micro-bulge, *h* is the distance between the micro-plane and the surface B, *J*_AWB_, *J*_AB_ are the Hamaker constant. As shown in Equations (8) and (9) [35]:

(8) $$J_{\mathrm{AWB}}=\left(\sqrt{J_{A}}-\sqrt{J_{W}}\right)\left(\sqrt{J_{B}}-\sqrt{J_{W}}\right)$$

(9) $$J_{\mathrm{AB}}=\sqrt{J_{A}}\sqrt{J_{B}}$$

where *J*_A_, *J*_W_, and *J*_B_ are the Hamaker constants of surface A, liquid, and surface B, respectively.

Combining the simplified solution method in reference [35], the van der Waals work of the micro-bulge with the base area an can be obtained by solving Equation (10):

(10) $$\left\{ \begin{array}{l} W_{c}=\frac{J_{\mathrm{AWB}}a_{n}^{D/2}}{12\pi hG^{D-1}}+W_{c1}\\ W_{c1}=\frac{\left(J_{\mathrm{AB}}-J_{\mathrm{AWB}}\right)a}{12\pi h^{2}} \end{array}\right.$$

For the micro-bulge without surface contact, the height of the micro-bulge is defined as *δ*_n_, and the height from the surface B to the top of the micro-bulge is *h* + *δ*_h_ − *δ*_n_, the research of Israelachvili gave the van der Waals force model between the ball and the plane [35], which can be obtained by Equation (11):

(11) $$W_{\mathrm{nc}}=\frac{J_{\mathrm{AWB}}a_{n}^{D/2}}{12\pi G^{D-1}\left[h+G^{D-1}\left(a_{L}^{1-D/2}-a_{l}^{1-D/2}-a_{n}^{1-D/2}\right)\right]}$$

Wang combined with the research results of Majumdar and Bhushan [21], proposed the expression of the number distribution of micro-bulge as [22]:

(12) $$n\left(a\right)=\frac{D}{2}\psi^{\left(2-D\right)/2}a_{L}^{D/2}a^{-\left(D+2\right)/2}$$

where *a*_L_ is the bottom area of the largest micro-bulge, and *ψ* is the extended domain factor of the distribution of micro-bulge, and its expression is:

(13) $$\frac{\psi^{\left(2-D\right)/2}-{\left(1+\psi^{-D/2}\right)}^{-(2-D)/D}}{\left(2-D\right)/D}=1$$

The rough surface is affected by van der Waals force, so the area where van der Waals force occurs is the apparent area *A*_a_, and its expression is shown in Equation (14). The real contact area of the deformed micro-bulge is *A*_r_, and the van der Waals force in this area shows the interaction of two planes, the expression is shown in Equation (15):

(14) $$A_{a}=\int_{0}^{a_{L}}an\left(a\right)da=\frac{D}{2-D}\psi^{\left(2-D\right)/2}a_{L}$$

(15) $$A_{r}=\int_{0}^{a_{l}}an\left(a\right)da=\frac{D}{2-D}\psi^{\left(2-D\right)/2}a_{l}$$

The ratio of the real contact area *A*_r_ to the apparent area *A*_a_ is:

(16) $$\frac{A_{r}}{A_{a}}=\frac{a_{l}}{a_{L}}$$

At the apparent area *A*_a_, the van der Waals work between surface A and surface B is:

(17) $$W=\int_{a_{h}}^{a_{L}}\left(W_{c}-W_{c1}\right)n\left(a_{n}\right)da_{n}+\int_{0}^{a_{h}}W_{\mathrm{nc}}\left(a_{n}\right)n\left(a_{n}\right)da_{n}+\int_{0}^{a_{l}}W_{c1}n\left(a\right)da$$

where *a*_h_ is the bottom area of the largest micro-bulge which is not in contact with surface B. Define *k* is the ratio of area *a*_l_ to *a*_L_, as *k* = *a*_l_/*a*_L_, therefore, the value of the critical contact area ratio *k*_pe_ for different materials can be obtained by Equation (18):

(18) $$k_{\mathrm{pe}}=\frac{a_{\mathrm{pe}}}{a_{L}}$$

When the micro-bulge is in the plastic deformation stage, according to studies of Majumdar [36], the force received by the micro-bulge in the plastic deformation stage is related to the hardness and the contact area. At this time, the relationship between the contact area ratio *k* and the pressure *P* is:

(19) $$P=H_{b}k$$

When the micro-bulge is in the stage of plastic deformation, the relationship between the contact area ratio *k* and the pressure *P*_e_ shows as Equation (20), and the expression of *a*_h_ shows in Equation (21):

(20) $$P_{e}=\frac{8G^{D-1}E^{*}k^{\frac{3-D}{2}}a_{L}{}^{\frac{3-D}{2}}}{3\pi^{1/2}}$$

(21) $$a_{h}={\left(\frac{\delta_{L}-\delta_{l}}{G^{D-1}}\right)}^{\frac{2}{2-D}}={\left(1-k^{\frac{2-D}{2}}\right)}^{\frac{2}{2-D}}a_{L}$$

The per unit area of Van der Waals force work *W’*(*h*) is shown in Equation (22):

(22) $$W^{\prime }\left(h\right)=\frac{J_{\mathrm{AWB}}\log{\left(\frac{a_{L}^{1-D/2}\left(1-k^{1-D/2}\right)}{h}\right)}^{2}}{24\pi G^{D-1}a_{L}^{1-D/2}\left(h+G^{D-1}a_{L}^{1-D/2}\left(1-k^{1-D/2}\right)\right)}+\frac{\left(J_{\mathrm{AB}}-J_{\mathrm{AWB}}\right)k}{12\pi h^{2}}+\frac{J_{\mathrm{AWB}}\log\frac{1}{1-k^{1-D/2}}}{12\pi hG^{D-1}a_{L}^{1-D/2}}$$

When a van der Waals force occurs on a non-flat rough surface, the van der Waals forces between surfaces are different from those on a flat rough surface due to the difference in peak and valley height of the surface contour. In this paper, an arc curve is used as the contour of the uneven rough surface, then the height difference of the uneven rough surface can be approximately characterized as flatness. The height difference of the non-flat rough surface is *H*, and the distance from a point on the surface to flat surface is *h’*, *s* is the area generating van der Waals force:

(23) $$ds=2\pi R_{A}dh^{\prime }$$

So, the van der Waals work between surface A and surface B is:

(24) $$W_{\mathrm{vdw}}=\int_{0}^{H}2\pi R_{A}W^{\prime }\left(h+h^{\prime }\right)dh^{\prime }$$

The van der Waals force between the two surfaces is:

(25) $$F_{\mathrm{vdw}}=\frac{dW_{\mathrm{vdw}}}{dh}=2\pi R_{A}\left(W^{\prime }\left(h+H\right)-W^{\prime }\left(h\right)\right)$$

### 3.3. Capillary Adhesion Force

**Figure 3:**
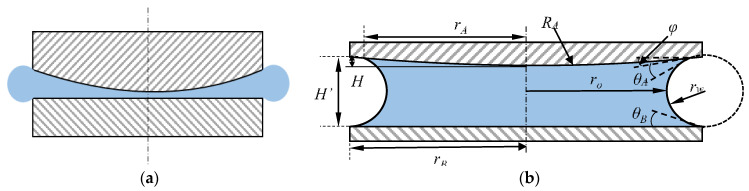
Capillary bridge between curved surface and flat surface: (**a**) the state in which the droplet is compressed between the two surfaces; (**b**) the two surfaces are separated and the droplets form a capillary bridge.

If there is a certain distance between the edges of the two surfaces, there is a pressure difference between atmospheric pressure and internal pressure in droplet, a meniscus between the two surfaces will be formed. Figure 3 shows the different states of droplet between to surface.

Petkov’s research found that the capillary bridge meniscus formed between the two plates exhibits an arc surface of radius *r* with a small amount of separation [37]. So the capillary force between the two surfaces can be expressed as:

(26) $$F_{\mathrm{cap}}=-\pi \left(2r_{o}\gamma_{s}\sin\varphi -r_{o}^{2}P_{c}\right)$$

where *γ*_s_ is the surface tension of the liquid in air, for water, *γ*_s_ = 72 × 10^−3^ N/m; *r*_o_ is the narrowest “neck” radius of the liquid bridge meniscus, and *θ*_A_ and *θ*_B_ are contact angle of non-flat rough surface A and surface B, respectively; *φ* is the angle between the surface tangent and the horizontal plane; *P*_c_ is the Laplace’s equation, and its expression is shown in Equation (27), which is related to the liquid surface tension *γ*_s_ and the radius of meniscus curvature *r*:

(27) $$P_{c}=\gamma_{s}\left(\frac{1}{r_{o}}+\frac{1}{r_{w}}\right)$$

Define *H’* as the maximum distance between the two surfaces of the capillary bridge, the expression of the curvature radius *r* and *r*_o_ of the curved surface is shown as:

(28) $$r_{w}=\frac{H^{\prime }}{\cos\left(\theta_{A}+\varphi \right)+\cos\theta_{B}}$$

(29) $$r_{o}=r_{B}-\frac{H^{\prime }\left(1-sin\theta_{B}\right)}{cos\left(\theta_{A}+\varphi \right)+cos\theta_{B}}$$

The expression of the capillary force between two surfaces is shown in Equation (30):

(30) $$F_{\mathrm{cap}}=\pi \gamma_{s}\left(r_{B}-\frac{H^{\prime }\left(1-sin\theta_{B}\right)}{cos\left(\theta_{A}+\varphi \right)+cos\theta_{B}}\right)\left[\frac{r_{B}\left(cos\left(\theta_{A}+\varphi \right)+cos\theta_{B}\right)-H^{\prime }\left(1-sin\theta_{B}\right)}{H^{\prime }}+\left(1-2\sin\varphi \right)\right]$$

If *r*_B_ is determined, the volume of the droplet determines the height *H’* of the curved surface when the maximum capillary force is generated. Assume *V* is the droplet volume, Equation (31) shows the relation between *V* and *H’*:

(31) $$\begin{array}{l} V\left(H^{\prime }\right)=\pi \left[{\left(r_{w}+r_{o}\right)}^{2}rp-\frac{\left(r_{w}+r_{o}\right)r_{w}{}^{2}}{2}\left(2\tan^{-1}\left(\cot\left(\theta_{A}+\varphi \right)\right)-sin2\left(\theta_{A}+\varphi \right)\right)+r_{w}{}^{3}\left(p^{2}-\frac{p^{3}}{3}\right)\right]\\ -\pi \left[{\left(r_{w}+r_{o}\right)}^{2}rq-\frac{\left(r_{w}+r_{o}\right)r_{w}{}^{2}}{2}\left(2\tan^{-1}\left(\cot\left(\theta_{B}\right)\right)-sin2\left(\theta_{B}\right)\right)+r_{w}{}^{3}\left(q^{2}-\frac{q^{3}}{3}\right)\right]-\pi R_{A}H^{2}+\frac{\pi }{3}H^{3} \end{array}$$

where

$$p=1+\cos\left(\theta_{A}+\varphi \right);\;q=1-\cos\theta_{B};\;R_{A}=\frac{r_{B}^{2}+H^{2}}{2H};\;\varphi =\arcsin\left(\frac{2Hr_{B}}{r_{B}^{2}+H^{2}}\right)$$

The contact angle is determined by factors such as material, surface roughness, droplet medium and air humidity. Under the conditions, according to Equation (32), it can be known that the macro contact angle *θ’* of the rough surface is related to the rough surface area ratio *ϕ* and the theoretical contact angle *θ*.

(32) $$1+\cos\theta^{\prime }=\phi \left(1+\cos\theta \right)$$

Assuming that the surface area of the micro-bulge before deformation is *s*_c_, then the value of *s* is shown in Equation (33):

(33) $$s_{c}=a_{n}+\pi G^{D-1}a_{n}^{2-D}$$

The surface area *S* of all the micro-convex bodies is shown in Equation (34):

(34) $$S=\int_{0}^{a_{L}}s_{c}n(a_{n})da_{n}=\frac{D\varphi^{\frac{2-D}{2}}a_{L}}{2-D}+\frac{\pi D\varphi^{\frac{2-D}{2}}}{4-3D}G^{2D-2}a_{L}^{2-D}$$

So, the area ratio of the rough surface *ϕ* is:

(35) $$\phi =\frac{s}{A_{a}}$$

The adhesion force between the two surfaces is:

(36) $$F=F_{\mathrm{vdw}}+F_{\mathrm{cap}}$$

## 4. Adhesion Force Experiment and Discussion of LSC

### 4.1. Experiment Preparation

**Figure 4:**
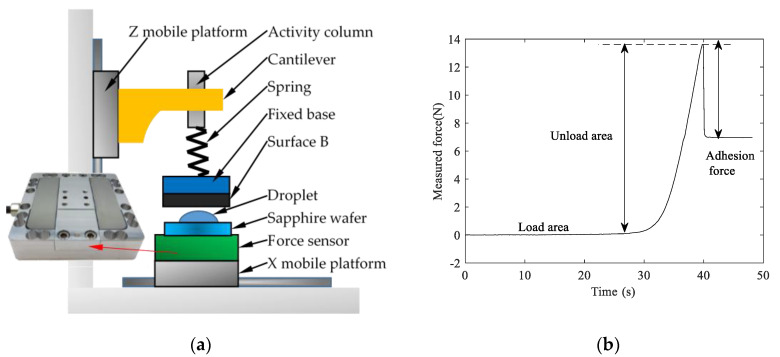
The test platform and the measurement curve structure: (**a**) structure diagram of triaxial force measuring platform; (**b**) the adhesion force measurement curve obtained by the test platform.

In order to verify the accuracy of the theoretical model, the adhesion force test was performed on the two adsorbed surfaces, the schematic diagram of the experimental device is shown in Figure 4a. The three-axis force sensor produced by ME-Meßsysteme is used to build a three-axis force measurement platform with a sampling frequency of 6.25 Hz and a measurement accuracy of 0.02 N. Figure 4b shows the force curve of the sensor obtained by the force measurement platform. It can be seen from the figure that there are three intervals in the measurement process, the first interval is the load area, and the second interval is the unload area, the third interval is the interval indicated by the adhesion force.

In this experiment, Sapphire wafer, aluminum alloy, iron, and 304 stainless steel were selected as the experimental objects, water was selected as the adhesion medium. The surface roughness *R*_a_, root mean square roughness (RMS), sampling frequency *ω* and other related parameters was obtained by Taylor Hobson’s surface profiler. Table 1 shows the parameters of different materials and roughness. Five different surface roughness of 304 stainless steel were measured to study the effect of roughness on the interface force.

Equation (37) shows the estimated relationship between fractal dimension and surface roughness *R*_a_ [38]:

(37) $$D=1.528R_{a}^{-0.042}$$

The relationship between the root mean square roughness (RMS) and the power spectrum is shown as:

(38) $$\mathrm{RMS}={\left(\int_{\omega_{l}}^{\omega_{h}}\frac{G^{2\left(D-1\right)}}{2\omega^{\left(5-2D\right)}\ln\gamma }d\omega \right)}^{1/2}$$

where *ω*_l_ is the cutoff frequency and *ω*_h_ is the high frequency which is determined by instrument resolution and filtering:

The analytical formula of *G* about fractal dimension *D* is shown in Equation (39) and Table 2 shows the Hamaker coefficient and other physical properties of different materials:

(39) $$G={\left(\frac{\left(4D-8\right){\mathrm{RMS}}^{2}\ln\gamma }{\omega_{h}^{\left(2D-4\right)}-\omega_{l}^{\left(2D-4\right)}}\right)}^{\frac{1}{2D-2}}$$

### 4.2. Results and Discussion

**Figure 5:**
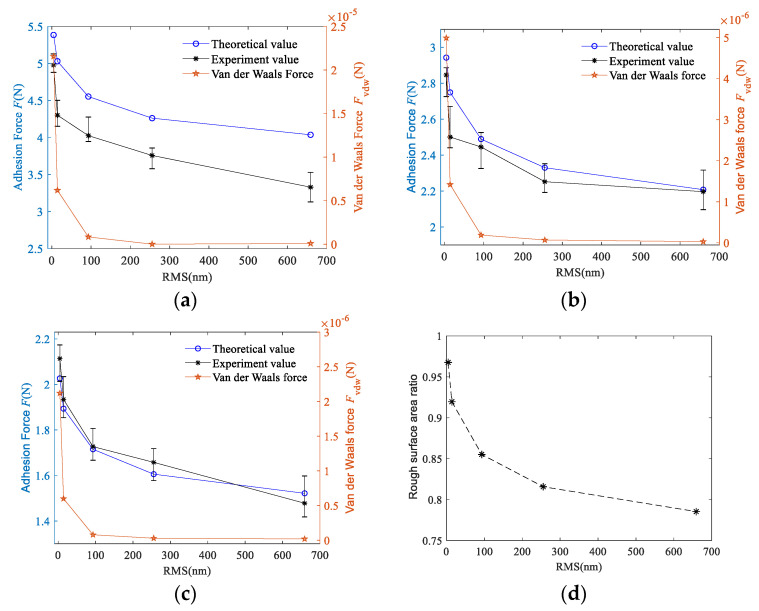
Effect of root mean square roughness (RMS) on adhesion force: (**a**) the droplet volume is 50 μL; (**b**) the droplet volume is 100 μL; (**c**) the droplet volume is 150 μL; (**d**) relationship between rough surface area ratio and RMS.

Figure 5 shows the relationship between the adhesion force and RMS when the load pressure is 0.8N. The theoretical and experimental adhesion forces obtained under the conditions of droplet volume of 50, 100, and 150 μL are shown in Figure 5a–c, respectively.

As can be seen from the change curve of adhesion force shown in Figure 5a, the adhesion force between the two surfaces showed a nonlinear downward trend. The change rule of the area ratio parameter *ϕ* is shown in Figure 5d. Area ratio parameter *ϕ* gradually decreases with the increase of roughness. According to Equation (32), the macro contact angle increases with the decrease of *ϕ*, the change is similar to the contact angle of 304 stainless steel with different roughnesses, as shown in Table 1. Therefore, in this experiment, the contact angle increases with the increase of roughness, which cause the decrease of capillary force. When the droplet has large volume, the distance between the two surfaces became larger, which makes the effect of van der Waals force decrease rapidly. At this point, the van der Waals force between the two surfaces is less than 10^−4^ N. When the surface roughness is decreased, the micro-bulge on the rough surface are smaller, which increases the van der Waals force between the single micro-bulge and the interface B. Therefore, the van der Waals force decreases with increasing roughness and gradually converges towards zero.

Figure 5b,c show the change curves of the adhesion force when the droplet volume is 100 and 150 μL, respectively. By comparing the values in Figure 5a to Figure 5c, it can be seen that under the same roughness, the difference in the adhesion force between different droplet volumes decreases with the increase of droplet volume. This difference between Figure 5a,b is greater than that of Figure 5b,c indicating that the adhesion force decreases non-linearly with the droplet volume increased. In addition, with the increase of droplet volume, the difference between the experimental value and the theoretical value gradually decreases, and even part of the experimental value in Figure 5c is greater than the theoretical value. This is because after the droplet volume increased, some droplets adhere to side of the substrate during the experiment and cannot participate in the formation of the capillary bridge. So, the height of the capillary bridge cannot reach the theoretical value.

Comparing Figure 5a to Figure 5c, it can be seen that the adhesion force between the two surfaces decreases with the increase of droplet volume. Combining Equation (31), it can be concluded that the maximum distance between the two surfaces of the capillary bridge *H’* increases when the volume of the droplet increases. As a result, “neck” radius *r*_o_ decreases and radius of meniscus curvature *r* increases. The orders of magnitude of *r*_o_ is much greater than *r*, so the orders of magnitude of 1/*r*_o_ increase is much smaller than 1/*r* decrease. The capillary pressure Pc between the two surfaces decreases, which leads to a decrease in the capillary bridge force *F*_cap_. This change can also be reflected in Equation (30).

**Figure 6:**
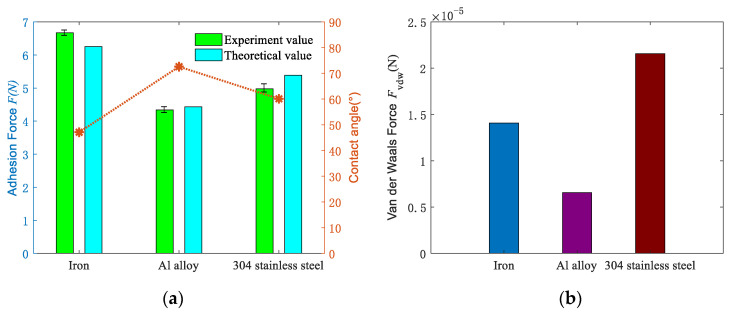
Influence of different materials on adhesion and van der Waals force: (**a**) comparison of experimental and theoretical values of adhesion force under different materials; (**b**) variation of van der Waals’ theoretical value under different materials.

Make the droplet volume is 50 μL, the effect of different materials on the adhesion force between the two surfaces was studied under approximate roughness conditions. Figure 6a shows the comparison of the theoretical value and experimental value of the adhesion force in different materials. It can be seen from the figure that the iron has the largest adhesion force on the sapphire wafer, followed by 304 stainless steel, and the Al alloy has the least. The red curve shows the contact angle of different materials, and its change rule is opposite to that of the adhesion force. It is also shown that the larger the contact angle is, the smaller adhesion force is.

Figure 6b shows the influence of different materials on the van der Waals force. The van der Waals force between different materials is mainly related to the Hamaker coefficient, the actual contact area and the distance between the two surfaces. When the distance is fixed, it is mainly determined by the hardness of the material and the Hamaker coefficient. It is proved that the van der Waals force of 304 stainless steel is the largest, the cast iron is the second, and the Al alloy is the smallest. Table 2 shows that Hamaker coefficient and hardness of the iron are higher than stainless steel, but the van der Waals force is lower than stainless steel. Equation (22) could explain the reason that the Hamaker coefficient decreases more obviously in the presence of medium, and its effect on van der Waals force is weaker than material hardness. The contact area ratio *k* is inversely proportional to the hardness. Therefore, the decrease in the contact area ratio caused by the increase in hardness makes the van der Waals force of iron smaller than that of stainless steel.

## 5. Double-sides Polishing Experiment Based on LSC

**Figure 7:**
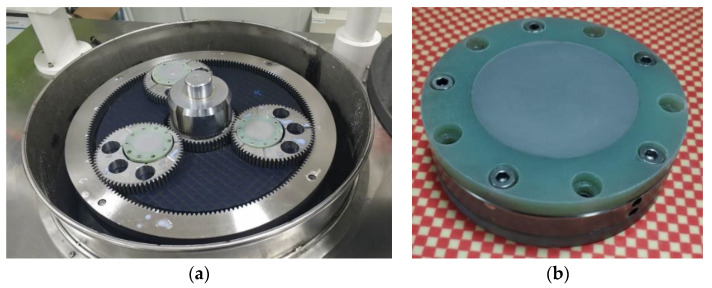
Layer stacked fixture and double-sides polishing equipment: (**a**) double-sides polishing machine; (**b**) layer stacked fixture.

According to the analysis, more than 1.4 N of normal adhesion force can be generated between sapphire wafer and baseplate. Sapphire wafer can be firmly adsorbed on the surface of baseplate, so the stacked clamping can be used for the double-sides processing of ultra-thin sapphire wafer. Comprehensive consideration of adhesion force and material characteristics, choose 304 stainless steel material for the baseplate, the experimental processing equipment and clamping method are shown in Figure 7.

The diameter of the sapphire wafer used in this experiment is Φ50.8 mm, the initial thickness is 0.43 mm. Before the double-sides polishing, the sapphire wafer was thinned by single-sided lapping, and the lapping slurry was prepared by Al_2_O_3_ powder with 3 μm particle size. Finally, an ultra-thin sapphire wafer with a thickness of 0.17 mm was obtained. The clamping thickness of the limiter was 0.105 mm. The specific experimental parameters are shown in Table 3.

**Figure 8:**
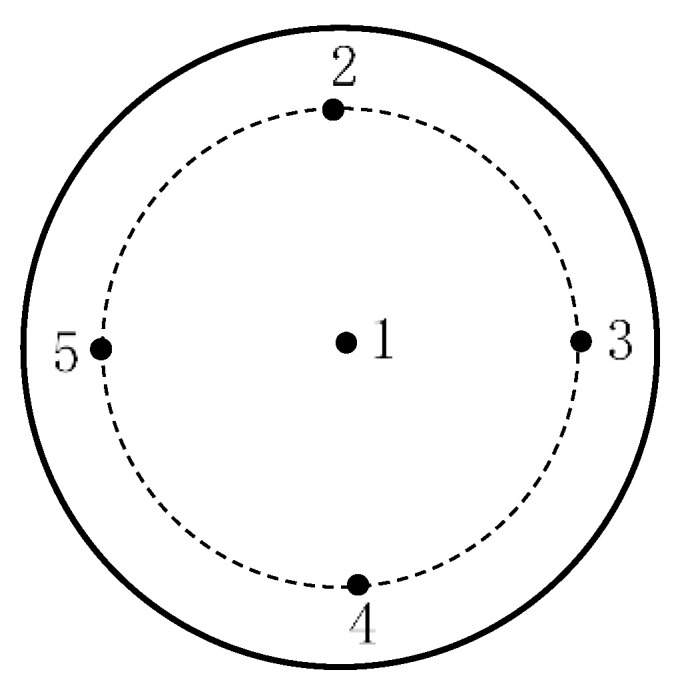
Schematic diagram of measuring points on sapphire wafer surface.

The surface roughness was used by the contact roughness tester produced by Taylor Hobson. Roughness measurement was performed every 60-min on the center point and another 4 points on the edge of sapphire surface, as shown in Figure 8. Each single point of each wafer was tested three times, the variation of roughness and its average value was obtained. The flatness of the final processed surface was measured using the GPI XP/D flatness meter produced by Zygo.

**Figure 9:**
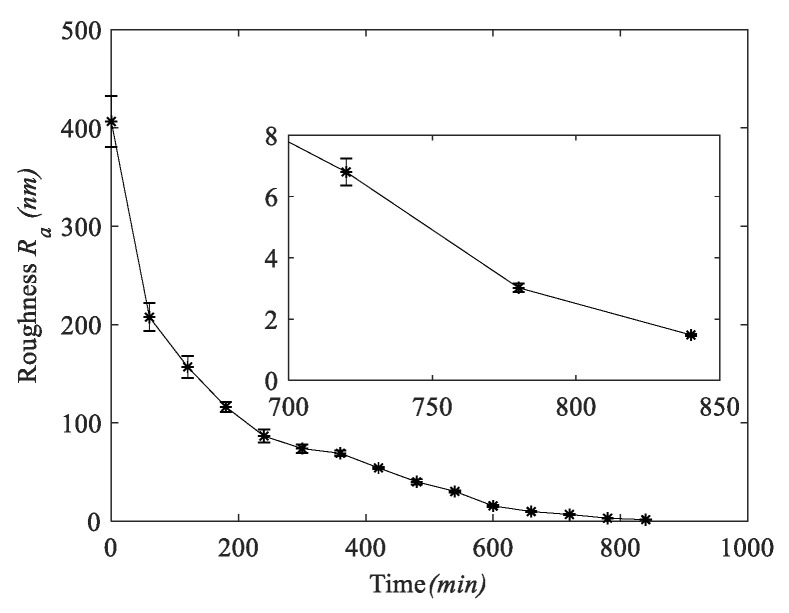
The change of surface roughness.

**Figure 10:**
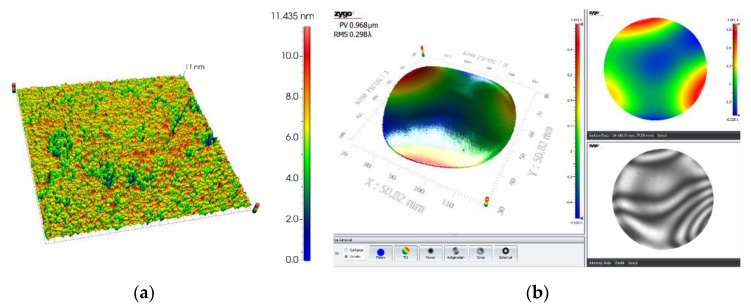
Surface morphology of sapphire based on LSC after double-sides polishing: (**a**) surface roughness measured by white light interferometer, the roughness (*R*_a_) is 1.4 nm and the 3D surface roughness (*S*_a_) is 1.1 nm; (**b**) the flatness of ultra-thin sapphire (PV) is 0.968 μm.

Figure 9 shows the variation curve of roughness with time in the experiment. It can be seen that the tendency of the amount of change in surface roughness decreases with increasing processing time, showing a non-linearly decreasing change. The error line of roughness also decreases with increasing processing time, indicating that the sapphire wafer surface has better uniformity and batch consistency during processing. As shown in Figure 10a, the surface morphology of the sapphire wafer photographed by the white light interferometer under the LSC method, the average surface roughness of sapphire is *R*_a_ = 1.52 nm, the optimal surface roughness (*R*_a_) is 1.4 nm, 3-D surface roughness (*S*_a_) is 1.1 nm. Figure 10b shows the flatness of ultra-thin sapphire wafers based on the double-sides polishing in the LSC mothed. The flatness (PV) can reach 0.968 μm.

From the experimental results of the LSC method, the problem of double-sides processing of ultra-thin sapphire wafers is effectively solved, and the high-precision of double-sides processing of ultra-thin sapphire wafers is realized.

## 6. Conclusion

In this work, the LSC method of ultra-thin sapphire wafer used in double-sides polishing was proposed and the clamping mechanism of LSC was researched. The following conclusions are obtained based on the experimental results:

1. Under the conditions of same pressure and surface spacing, the van der Waals force is mainly determined by hardness and Hamaker coefficient of material.
2. The adhesion force between the solid-liquid interface is mainly depends on capillary force, and van der Waals force is almost negligible.
3. The effect of capillary force is mainly affected by the volume of droplet, roughness and material. With the increasing of droplet volume, the height of completely capillary bridge formed between the two surfaces will also increasing, and the roughness and material will affect the contact angle of the surface.
4. Through the LSC method, the ultra-thin sapphire wafer can obtain an average surface roughness (*R*_a_) of 1.52 nm and a flatness (PV) of 0.968 μm.

The LSC method is capable for double-sides ultra-thin sapphire wafer polishing, which may have great potentials in ultra-thin wafer processing.

## Figures and Tables

**Table 1:** Surface parameters of different materials.

Material	Sapphire	Al Alloy	Iron	304 Stainless Steel				
				1	2	3	4	5
*R*_a_ (nm)	3.5	6.5	6.4	3.6	12.1	68.2	210.1	517.9
Root mean square roughness (RMS) (nm)	4.2	8.2	7.8	4.4	14.2	93.0	255.4	659.0
Connect angle *θ’* (°)	51.2	72.6	47.1	60.1	66.7	82.6	84.8	86.5
Cutoff frequency *ω*_l_	12.5	12.5	12.5	12.5	12.5	4	1.25	1.25
High frequency *ω*_h_	400							
Height difference *H’* (μm)	-	10						

**Table 2:** Physical properties of different material.

Parameter	Material				
	Water	Sapphire	Iron	Al Alloy	304 Stainless Steel
Hamaker coefficient *J* (10^−20^ J)	3.7	15.5	26	12.6	21.2
Elastic Modulus (GPa)	-	379	210	68.9	193
Poisson’s ratio	-	0.309	0.3	0.33	0.29
Brinell hardness *H*_b_ (N/mm^2^)	-	-	146	30	123

**Table 3:** Polishing experiment parameters.

Name	Parameter	Name		Parameter
Sapphire	α-Al_2_O_3_ C direction	Rotation	Upper plate (r/min)	−24
Diameter of sapphire (mm)	Φ50.8		Lower plate (r/min)	34
Sapphire thickness (mm)	0.17		Sun gear (r/min)	20
Abrasive	SiO_2_		Outer gear (r/min)	0
Abrasive size (nm)	80	pH of slurry		11
Flow rate of slurry (mL/min)	25	Flatness of baseplate (μm)		0.988
Quality score of slurry (%wt)	5	Thickness of limit tablet (mm)		0.105
Pressure (KPa/piece)	31.6	Time (min)		60

## References

[1] Nakamura S., Senoh M., Iwasa N. (1995). High-brightness InGaN blue, green and yellow light-emitting diodes with quantum well structures. Jpn. J. Appl. Phys..

[2] Nakamura S., Senoh M., Nagahama S.I., Iwasa N., Yamada T. (1996). Characteristics of InGaN multi-quantum-well-structure laser diodes. Appl. Phys. Lett..

[3] Wang T., Bai J., Sakai S. (2001). Influence of InGaN/GaN quantum-well structure on the performance of light-emitting diodes and laser diodes grown on sapphire substrates. J. Cryst. Growth.

[4] Ploch N.L., Rodriguez H., Stolmacker C., Hoppe M., Lapeyrade M., Stellmach J., Mehnke F., Wernicke T., Knauer A., Kueller V. (2013). Effective thermal management in ultraviolet light-emitting diodes with micro-LED arrays. IEEE Trans. Electron Devices.

[5] Gong Z., Jin S., Chen Y., McKendry J., Massoubre D., Watson I.M., Gu E., Dawson M.D. (2010). Size-dependent light output, spectral shift, and self-heating of 400 nm InGaN light-emitting diodes. J. Appl. Phys..

[6] Vitusevich S.A., Kurakin A.M., Klein N., Petrychuk M.V., Naumov A.V., Belyaev A.E. (2008). AlGaN/GaN high electron mobility transistor structures: Self-heating effect and performance degradation. IEEE Trans. Device Mater. Reliab..

[7] Horng R.-H., Wuu D.-S., Lin C.-F., Lai C.-F. (2018). Recent development of fabrication technologies of nitride LEDs for performance improvement. Nitride Semiconductor Light-Emitting Diodes (LEDs).

[8] Kumar P., Lee J., Lee G., Rao S., Singh D., Singh R.K. (2013). Low temperature wet etching to reveal sub-surface damage in sapphire substrates. Appl. Surf. Sci..

[9] Wan L., Dai P., Li L., Deng Z., Hu Y. (2019). Investigation on ultra-precision lapping of A-plane and C-plane sapphires. Ceram. Int..

[10] Zhao D., Xu S., Xie M., Tong S., Yang H. (2003). Stress and its effect on optical properties of GaN epilayers grown on Si (111), 6H-SiC (0001), and c-plane sapphire. Appl. Phys. Lett..

[11] Sheng H., Hang W., Chen Z. (2019). Effect of Surface Roughness of Stainless-steel Substrate on Water-film Adhesion. Surf. Technol..

[12] Goto K., Mochiji K., Moritani K., Inui N. (2014). Roughness Dependence of the Casimir Force between Fractal Surfaces. e-J. Surf. Sci. Nanotechnol..

[13] Carrion-Vilches F.J., Bermudez M.D., Fructuoso P. (2015). Static and kinetic friction force and surface roughness of different archwirebracket sliding contacts. Dent. Mater. J..

[14] Liou J.L., Lin J.F. (2007). A new microcontact model developed for variable fractal dimension, topothesy, density of asperity, and probability density function of asperity heights. ASME.

[15] Morag Y., Etsion I. (2007). Resolving the contradiction of asperities plastic to elastic mode transition in current contact models of fractal rough surfaces. Wear.

[16] Liou J.L., Lin J.F. (2010). A modified fractal microcontact model developed for asperity heights with variable morphology parameters. Wear.

[17] Liou J.L., Tsai C.M., Lin J.F. (2010). A microcontact model developed for sphere- and cylinder-based fractal bodies in contact with a rigid flat surface. Wear.

[18] Miao X.M., Huang X.D. (2014). A complete contact model of a fractal rough surface. Wear.

[19] Greenwood J.A., Williamson J.B.P.P. (1966). Contact of Nominally Flat Surfaces. Proc. R. Soc. Lond..

[20] Hertz H. (1896). Über die berührung fester elastischer Körper (On the contact of rigid elastic solids). J. Reine Und Angew. Math..

[21] Majumdar A., Bhushan B. (1991). Fractal Model of Elastic-Plastic Contact Between Rough Surfaces. ASME.

[22] Wang S., Komvopoulos K. (1994). A Fractal Theory of the Interfacial Temperature Distribution in the Slow Sliding Regime: Part II—Multiple Domains, Elastoplastic Contacts and Applications. J. Tribol..

[23] Thom C., Brodsky E.E., Goldsby D.L., Candela T., Carpick R.W. (2015). Nanoscale Characterization of Fault Roughness by Atomic Force Microscopy. AGUFM.

[24] Gurdogan E.B., Ozdemir-Ozenen D., Sandalli N. (2017). Evaluation of Surface Roughness Characteristics Using Atomic Force Microscopy and Inspection of Microhardness Following Resin Infiltration with Icon. J. Esthet. Restor. Dent..

[25] Voïtchovsky K., Kuna J.J., Contera S.A., Tosatti E., Stellacci F. (2010). Direct mapping of the solid–liquid adhesion energy with subnanometre resolution. Nat. Nanotechnol..

[26] Terriza A., Alvarez R., Yubero F., Borras A., González-Elipe A.R. (2011). Comments on “An Essay on Contact Angle Measurements”: Determination of Surface Roughness and Modeling of the Wetting Behavior. Plasma Process. Polym..

[27] Wang Y., Michielsen S., Lee H.J. (2013). Symmetric and asymmetric capillary bridges between a rough surface and a parallel surface. Langmuir.

[28] Hongbo Z., Huang J., Tian Y., Li L., Tirrell M.V., Israelachvili J.N. (2016). Adhesion and Detachment Mechanisms between Polymer and Solid Substrate Surfaces: Using Polystyrene–Mica as a Model System. Macromolecules.

[29] Hiep N.H., Thanh M.D., Huy N.D. (2018). Viscous–capillary traveling waves associated with classical and nonclassical shocks in van der Waals fluids. Nonlinear Anal. Real World Appl..

[30] Harrison J.A. (2015). Detailed Investigations of Capillary and van der Waals Forces in the Adhesion between Solids. Ph.D. Thesis.

[31] Qing T., Shao T.-m., Wen S.-z. (2006). Effects of relative humidity on surface adhesion. Tribology.

[32] Yastrebov V.A., Anciaux G., Molinari J.F. (2015). From infinitesimal to full contact between rough surfaces: Evolution of the contact area. Int. J. Solids Struct..

[33] Beamer B.S., Walley K.C., Okajima S., Manoukian O.S., Perez-Viloria M., DeAngelis J.P., Ramappa A.J., Nazarian A. (2017). Changes in Contact Area in Meniscus Horizontal Cleavage Tears Subjected to Repair and Resection. Arthrosc. J. Arthrosc. Relat. Surg..

[34] Jacobs T.D.B., Martini A. (2017). Measuring and Understanding Contact Area at the Nanoscale: A Review. Appl. Mech. Rev..

[35] Israelachvili J.N. (2011). Intermolecular and Surface Forces: Revised Third Edition.

[36] Tan T.H., Yan J. (2017). Atomic-scale characterization of subsurface damage and structural changes of single-crystal silicon carbide subjected to electrical discharge machining. Acta Mater..

[37] Petkov P.V., Radoev B., Kliment S., Blvd J.B. (2019). Investigation of Single and Binary of “Sandwich” Type Convex Liquid Capillary Bridges, Stretched between Two Flat Surfaces (Experimental Approach). Colloids Interfaces.

[38] Ge S., Tonder K. (1997). The fractal behavior and fractal characterization of rough surfaces. Tribology.

